# Diagnostic Issues in Other Mental Disorders Co-Morbid With Intellectual Disability

**DOI:** 10.1192/j.eurpsy.2023.90

**Published:** 2023-07-19

**Authors:** J. P. Albuquerque

**Affiliations:** Centro Recuperação Menores, Instituto Irmãs Hospitaleiras Sagrado Coração Jesus, Portalegre, Portugal

## Abstract

**Abstract:**

The assumption by ICD-11 of Intellectual Disability (ID) as part of the Neurodevelopmental Disorders, named Intellectual Developmental Disorders (IDD), bring more attention of the psychiatric community to this population and the problems they face.

In the field of neurodevelopmental disorders, different diagnosis intersect, since they share symptoms, and some of those symptoms are difficult to define.

At the same time, the difficulty of people with ID to conceptualize and / or express is subjective experience creates another obstacle to clearly identify witch symptoms are present.

We will try to discuss these questions, and offer some references, in order to help mental health professionals to recognize the most common diagnosis in people with ID, and differentiate between them, namely Autism Spectrum Disorder, Attention-Deficit Hyperactivity Disorder, Mood Disorders, and Impulse Control Disorders.

**Disclosure of Interest:**

None Declared

